# CD200 expression is a feature of solid pseudopapillary neoplasms of the pancreas

**DOI:** 10.1007/s00428-018-2437-7

**Published:** 2018-08-21

**Authors:** Rita T. Lawlor, Valentina Daprà, Ilaria Girolami, Antonio Pea, Camilla Pilati, Alessia Nottegar, Paola Piccoli, Claudia Parolini, Nicola Sperandio, Paola Capelli, Aldo Scarpa, Claudio Luchini

**Affiliations:** 1 ARC-Net Research Center, University of Verona, Verona, Italy; 2 Department of Diagnostics and Public Health, Section of Pathology, University and Hospital Trust of Verona, Piazzale Scuro, 10, 37134 Verona, Italy; 3 Department of General and Visceral Surgery, Pancreas Institute, University and Hospital Trust of Verona, Verona, Italy; 4 Personalized Medicine, Pharmacogenomics, Therapeutic Optimization, Paris-Descartes University, Paris, France; 5 Department of Surgery, Section of Pathology, San Bortolo Hospital, Vicenza, Italy

**Keywords:** SPN, SPT, Acinar carcinoma, CD200, Immunohistochemistry, Pancreatic cancer

## Abstract

CD200 has been recently indicated as a robust marker of well-differentiated neuroendocrine neoplasms. Here, we evaluate its role in differential diagnosis of solid pancreatic neoplasms. We immunostained for CD200 22 solid pseudopapillary neoplasms (SPNs), 8 acinar carcinomas (ACs), 2 pancreatoblastomas (PBs), 138 neuroendocrine tumors (PanNETs), and 48 ductal adenocarcinomas. All SPNs showed strong cytoplasmic and membranous staining for CD200, while only one case of AC had focal positivity. The two PBs showed focal CD200 positivity, mainly located in squamoid nests. The vast majority of PanNETs (96%) showed strong cytoplasmic and membranous staining for CD200, whereas all PDACs were negative. As both PanNETs and SPNs express CD200, it has no role in the differential diagnosis between these two entities.

## Introduction

CD200 is a membrane protein, member of the type I immunoglobulin superfamily [1, 2]. It is normally expressed by neurons, endothelial cells, and several types of inflammatory cells including lymphocytes [3]. CD200 expression has been documented in different tumor types, like colorectal and ovarian cancers [4–6]. In some neoplasms, CD200-positive tumor cells exhibit cancer stem cell properties, capable of self-renewal and displaying pluripotent potential [4, 6].

Notably, a recent report has shown that CD200 is expressed by neuroendocrine neoplasms of any anatomical site, including pancreatic neuroendocrine tumors (PanNETs) [3]. In that study, CD200 was positive in 94% of 60 PanNETs against none of five pancreatic ductal adenocarcinomas (PDAC) studied. In particular, it was widely expressed with a strong positivity and a staining pattern that was both membranous and cytoplasmic [3] in 100% (37/37) of well-differentiated G1 PanNETs and 83% (19/23) of moderately differentiated G2 PanNETs [3]. Given that the study was aimed at evaluating neuroendocrine neoplasms of various organs, it did not evaluate other pancreatic neoplasms to provide a complete differential diagnosis for neoplasms of this organ.

As the discovery of new immunohistochemical markers is of particular interest for PanNETs, we decided to explore CD200 expression in pancreatic tumors that represent their most important differential diagnosis: solid pseudopapillary neoplasms (SPNs), acinar cell carcinomas (ACs), and pancreatoblastomas (PBs).

## Materials and methods

This study was approved by the Local Ethics Committee (Prog.1743CESC, 18/04/2018). Twenty-two SPNs, 8 ACs, 2 PBs, 138 PanNETs, and 48 PDAC were retrieved from the ARC-Net Biobank of the University and Hospital Trust of Verona. The 138 PanNETs were arranged in 6 tissue micro-arrays (TMAs, 3 cores per case). The 48 PDACs were arranged in 2 TMAs, each containing 24 cases (4 cores per case).

Immunohistochemistry for CD200 (R&D Systems, clone: XP_005548207, dilution 1:100), chromogranin-A (Dako, clone: DAK-A3, dilution 1:2500), synaptophysin (Novocastra, clone: 27G12, dilution 1:100), and β-catenin (Sigma-Aldrich, clone: 15B8, dilution 1:400) was performed as previously described [7]. Briefly, 4-μm formalin-fixed paraffin-embedded sections were used. Heat-induced antigen retrieval was performed using a heated plate and 0.01 mol/l of citrate buffer, pH 8.9, for 15 min. Light nuclear counterstaining was performed with hematoxylin (5 min). All samples were processed using a sensitive peroxidase-based “Bond polymer Refine” detection system (Vision-Biosystem, Leica, Milan, Italy). Blinded evaluation on the immunohstochemical slides was performed by three gastrointestinal pathologists (C.L., P.C., A.S.).

An integrated score (IS) was used to report CD200 immunostaining by multiplying the value attributed to the percentage of positive cells (0% = score 0, 1–25% = score 1, 26–50% = score 2, 51–75% = score 3, 76–100% = score 4) with the value assigned to the intensity of staining (absent = score 0, weak = score 1, moderate = score 2, strong = score 3). Thus, the IS obtained ranged from 0 to 12. The cellular compartment stained was also reported: membranous, cytoplasmic, or nuclear.

## Results

The results of immunostaining for CD200 are summarized in Table 1.

**Table 1:** Summary of the results of CD200 immunohistochemistry

Antibody	SPN		AC		PanNET		PDAC		PB	
	No. of positive cases	IS in positive cases	No. of positive cases	IS in positive cases	No. of positive cases	IS in positive cases	No. of positive cases	IS in positive cases	No. of positive cases	IS in positive cases
CD200	22/22 (100%)	12	1/8 (12.5%)	2	132/138 (96%)	12	0/48 (0%)	0	2/2(100%)	2

*SPN*, solid pseudopapillary neoplasm; *AC*, acinar cell carcinoma; *PanNET*, pancreatic neuroendocrine tumor; *PDAC*, pancreatic ductal adenocarcinoma; *PB*, pancreatoblastoma

IS stands for integrated score, which takes into account both the distribution and the intensity of the immunohistochemical staining (see the “Materials and methods” section)

**Fig. 1:**
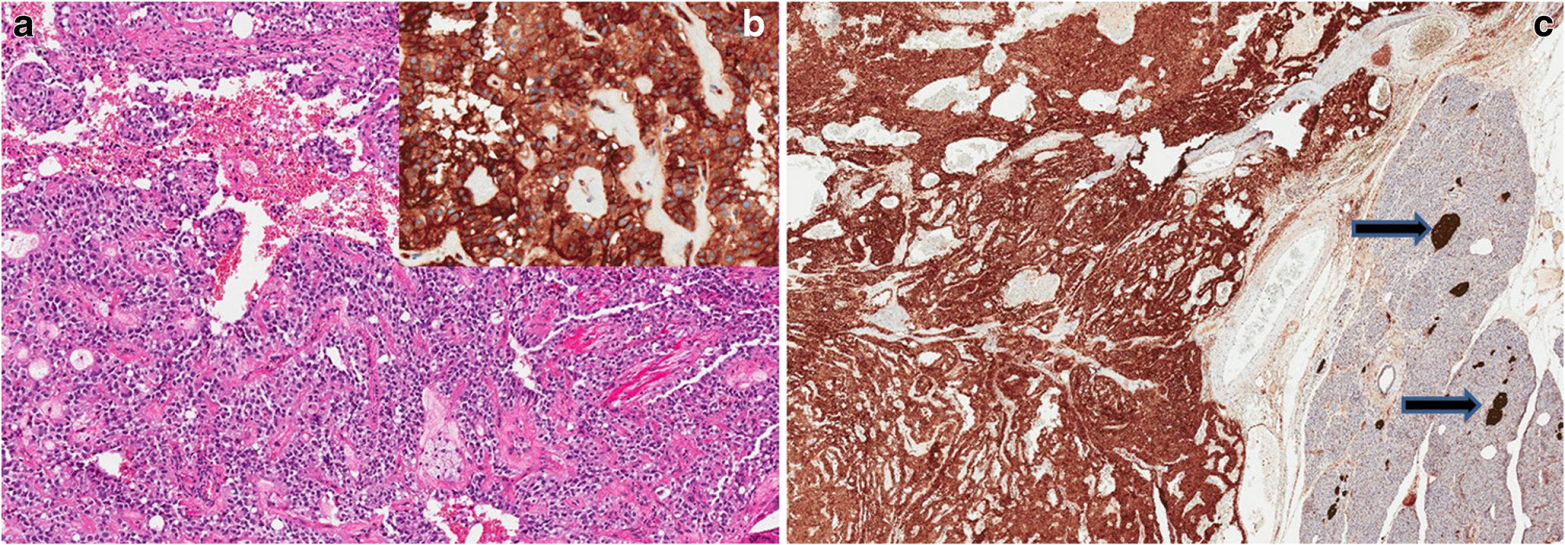
A paradigmatic case of solid pseudopapillary neoplasm is shown. **a** The typical morphology of SPN has some morphological/architectural aspects similar to PanNET, such as the uniform cell size and the intimate vascular permeation (hematoxylin and eosin, original magnification × 10). **b** Immunohistochemistry for CD200 with strong membranous and cytoplasmic positivity in all tumor cells, and negative nuclei (original magnification × 20). **c** CD200 immunostaining showing diffuse positivity of tumor cells and Langerhans islets which are a positive internal controls (arrows) (original magnification × 4)

All the 22 SPNs were positive for CD200 with an IS of 12. All neoplastic cells showed a strong positivity characterized by both cytoplasmic and membranous staining (Fig. 1).

**Fig. 2:**
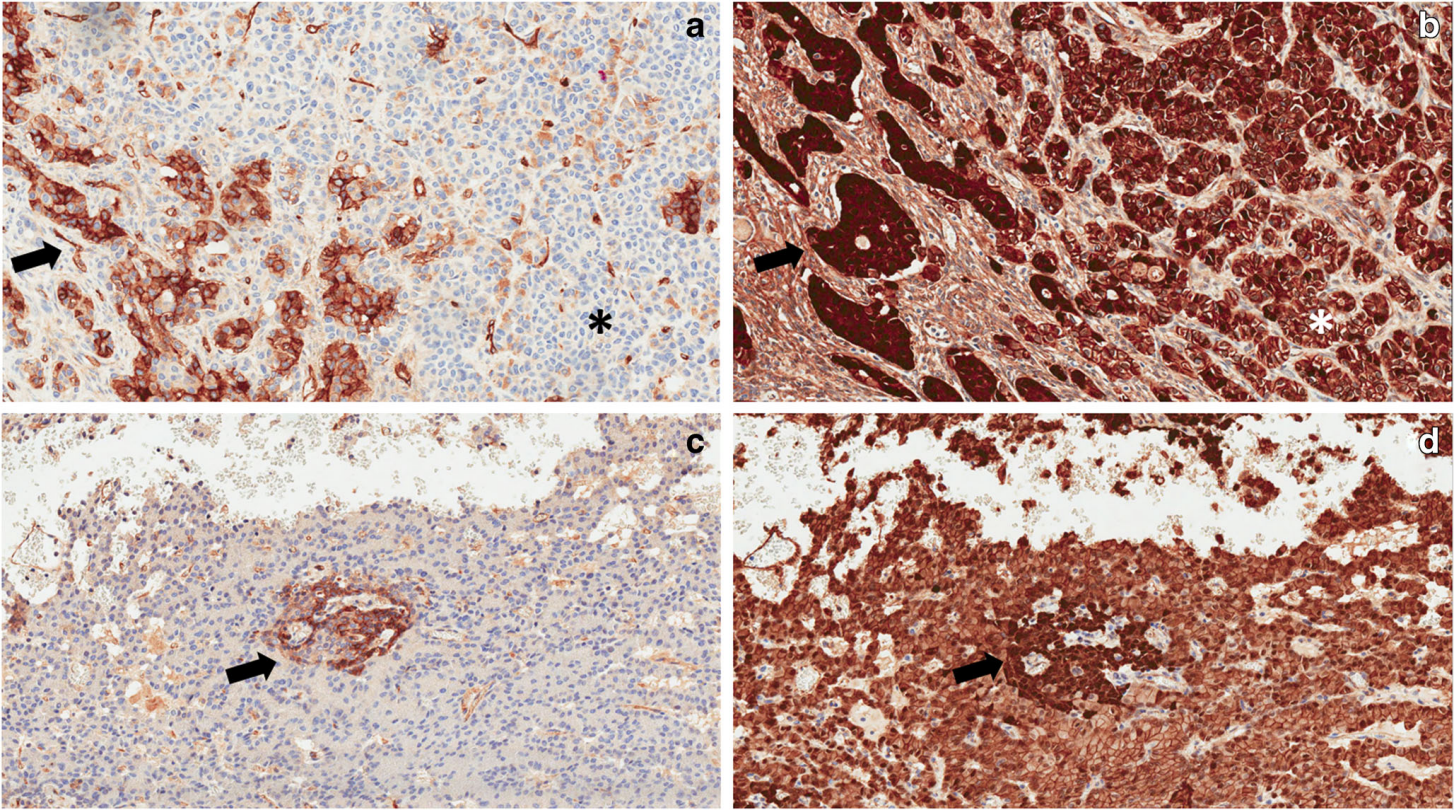
**a** The case of acinar cell carcinoma with focal CD200 is shown: in a negative background, there are few cells and cell clusters with a moderate CD200 positivity of both cytoplasm and cell membrane. Nuclei are always negative (original magnification × 20). **b** β-Catenin staining (same area of **a**): all cells present a membranous positivity for β-catenin, since this protein is constitutively expressed in the membrane of all cell types; note that there is a clear correspondence between CD200 and nuclear β-catenin immunostaining (black arrows, original magnification × 20). The cells that are CD200 negative present only a membranous staining pattern for β-catenin (asterisks). **c** CD200 expression pattern in a case of pancreatoblastoma; positive cells are mainly located in a squamoid nest (black arrow, original magnification × 20). **d** β-Catenin staining (same area of **c**): there is a clear correspondence between CD200 and nuclear β-catenin immunostaining also in this case (black arrow, original magnification, × 20)

Only 1 of the 8 ACs (12.5%) showed, on a negative background, scattered clusters of cells with a membranous and/or cytoplasmic CD200 immunostaining (Fig. 2a, b). The percentage of positive cells was about 15%, and the intensity of staining was moderate, resulting in an IS of 2. All other ACs showed no CD200 expression (IS = 0).

PBs showed focal membranous and/or cytoplasmic positivity for CD200, mainly in squamoid nests (Fig. 2c, d). The percentage of positive cells was 15–20% in both cases, and the intensity of staining was moderate, resulting in an IS of 2.

Of the 138 PanNETs, all 84 G1 cases (100%) and 48 of the 54 G2 cases (89%) showed strong cytoplasmic and membranous staining for CD200 of all neoplastic cells with an IS of 12.

All 48 PDAC cases were negative for CD200 immunostaining (IS = 0), in the presence of CD200 positively staining internal controls, comprising Langerhans islets, endothelial cells and nerves, and Pacinian bodies found in some cases in the peri-pancreatic adipose tissue (3).

Chromogranin-A (IS = 0) was negative in all cases of SPNs, ACs, and PBs, while synaptophysin was positive in 10 of 22 (45%) SPNs with a focal (10–20% of positive cells) and moderate positivity for CD200 (membranous pattern, IS = 2). Of the ACs and PBs, no case showed positivity for synaptophysin (IS = 0).

β-Catenin showed nuclear staining in all 22 SPNs, 1 AC (the one with focal CD200 positivity), and 2 PBs. Notably, the β-catenin nuclear localization in the positive AC and PBs was restricted to the CD200-positive isolated cells or cell clusters (Fig. 2b, d).

## Discussion

In this work, we report that the expression of CD200 is a feature of pancreatic SPNs, all cases showing a strong cytoplasmic and membranous immunostaining in all neoplastic cells. We found only 1 case of AC with focal positivity for CD200, having some isolated cells and cell clusters a cytoplasmic and/or a membranous staining pattern. The vast majority of PanNETs were positive for CD200 with a strong cytoplasmic and membranous immunostaining in all neoplastic cells, whereas no PDAC presented any staining.

Such results may have important implications, first of all for routine histopathological activity. In fact, a recent study pointed out that neuroendocrine tumors show strong immunostaining for CD200 [3], and suggested that this marker was very sensitive for PanNET, with complete sensitivity (100%) for G1 and high sensitivity (83%) for G2 cases [3]. We confirm in our cohort of 138 PanNETs the complete sensitivity for G1 and high (89%) sensitivity for G2. We also confirm the lack of expression of CD200 in PDAC on a large series of 48 cases, as Love et al. only included 5 cases in their study. Considering that SPNs, ACs, and PBs represent the pancreas neoplasms that undergo differential diagnosis from PanNET, the description of the CD200 positivity in pancreatic SPNs with the same pattern described for PanNET is relevant. Indeed, SPNs may be misdiagnosed as PanNET [8–10], and our data demonstrate that CD200 marker is of no help in such differential diagnosis. We also confirm that synaptophysin, a classic neuroendocrine marker, is very often positive in SPNs (45% of the cases in our cohort) [11]. Moreover, CD200 positivity does not permit the exclusion of a diagnosis of AC, as 1 out of 8 cases of AC in our cohort displayed focal positivity.

The diffuse and strong immunoreactivity of CD200 in SPNs may provide some insights into the biology of this peculiar tumor entity. A seminal paper of Kosmahl et al. on this topic highlighted that the only biological connection of SPN was with neuroendocrine differentiation, due to the presence of synaptophysin positivity in a significant percentage of cases [11]. Notably, also NSE and CD56 neuroendocrine markers are shared by PanNETs and SPN [12, 13]. In view of the striking female preponderance of SPNs and of the known close proximity of the genital ridges to the pancreatic anlage during embryogenesis, Kosmahl et al. suggested that SPNs might derive from genital ridge/ovarian anlage-related cells, which are in contact with the pancreatic tissue during early embryogenesis [11]. Our data could be in line with this hypothesis as CD200 is a marker associated not only with neuroendocrine differentiation but also with ovarian neoplasms [5, 11], highlighting the potential importance of embryogenesis in SPN origin. Since CD200 has been indicated as a marker of stem cell status, its strong and diffuse expression in both PanNETs and SPNs may support their biological association.

Whole exome sequencing has highlighted that SPNs contain remarkably few genetic alterations in addition to the constantly present *CTNNB1* mutation, a key component of the Wnt pathway [14]. This latter is responsible for the constitutive activation of β-catenin, which is identified by its pathognomonic nuclear localization. Notably, a strong association between CD200 expression and activation of Wnt/β-catenin pathway has been recently described in colorectal cancer [6]. The positivity of CD200 in SPNs confirms this correlation, since all tumor cells of our SPN cohort had nuclear β-catenin. Strikingly, the case of AC with focal CD200 staining was the only AC with β-catenin nuclear immunostaining that was restricted to the very same CD200-positive cells and cell clusters, further corroborating the intimate correlation between CD200 and Wnt/β-catenin pathway. A similar situation was observed for PBs, in which CD200 and nuclear β-catenin were expressed by the same cells (mainly cells of the squamoid nests).

In conclusion, we describe for the first time CD200 expression in SPNs and ACs, and therefore its immunohistochemical expression is of no utility in the differential diagnosis among PanNET, SPN, or AC. The strong and diffuse expression of this marker with an identical cytoplasmic and membranous pattern in PanNETs and SPNs may support the existing hypothesis of biological similarities between these two pancreatic tumor entities.
